# Reduction in kidney function decline and risk of severe clinical events in agalsidase beta–treated Fabry disease patients: a matched analysis from the Fabry Registry

**DOI:** 10.1093/ckj/sfae194

**Published:** 2024-07-02

**Authors:** Julie L Batista, Ali Hariri, Manish Maski, Susan Richards, Badari Gudivada, Lewis A Raynor, Elvira Ponce, Christoph Wanner, Robert J Desnick

**Affiliations:** Epidemiology/Biostatistics, Sanofi, Cambridge, MA, USA; Clinical Development and Medical Affairs, LG Chem Life Sciences, Boston, MA, USA; Global Medical Affairs, Rare Nephrology, Sanofi, Cambridge, MA, USA; Global Medical Affairs, Rare Nephrology, Sanofi, Cambridge, MA, USA; Translational Medicine and Early Development, Sanofi, Cambridge, MA, USA; Epidemiology/Biostatistics, Sanofi, Cambridge, MA, USA; Epidemiology/Biostatistics, Sanofi, Cambridge, MA, USA; Epidemiology, Biogen, Cambridge, MA, USA; Global Medical Affairs, Rare Nephrology, Sanofi, Cambridge, MA, USA; Division of Nephrology, Department of Medicine, University Hospital Würzburg, Würzburg, Germany; Department of Genetics and Genomic Sciences, Icahn School of Medicine at Mount Sinai, New York, NY, USA

**Keywords:** agalsidase beta, composite clinical event, Fabry disease, kidney function, matched analysis

## Abstract

**Background:**

Patients with Fabry disease (FD, α-galactosidase A deficiency or absence) accumulate glycosphingolipids, leading to progressive dysfunction of kidneys, heart and nervous system. Generalizable real-world outcomes following agalsidase beta treatment initiation outside trials are limited. We investigated the associations of long-term agalsidase beta treatment with estimated glomerular filtration rate (eGFR) changes over time and the risk of developing a composite clinical event in a matched analysis of treated and untreated patients with FD.

**Methods:**

Agalsidase beta–treated adult patients (aged ≥16 years) from the Fabry Registry and adult untreated patients from a natural history cohort were matched 1:1 and X:X (with one occurrence and multiple occurrences of each untreated patient, respectively) by sex, phenotype, age and (for eGFR slope analysis) baseline eGFR. Outcomes included eGFR slope over 5 years and composite clinical event risk (cardiovascular, cerebrovascular or renal event, or death) over 10+ years. As a surrogate indicator of therapeutic response in paediatric patients, the percentage experiencing normalization in plasma globotriaosylceramide (GL-3) from treatment initiation was assessed in patients aged 2 to <16 years.

**Results:**

Overall, eGFR slopes for 1:1-matched untreated and treated adult patients [122 pairs (72.1% male)] were −3.19 and −1.47 mL/min/1.73 m^2^/year, respectively (reduction in rate of decline = 53.9%, *P* = .007), and for X:X-matched [122 untreated/950 treated (59.4% male)] were −3.29 and −1.56 mL/min/1.73 m^2^/year, respectively (reduction in rate of decline = 52.6%, *P* < .001). Agalsidase beta treatment was associated with lower risk of clinical events, with hazard ratios of 0.41 (*P* = .003) and 0.67 (*P* = .008) for 1:1-matched and X:X-matched analyses, respectively. Plasma GL-3 declined markedly in paediatric patients and normalized in most within 6 months of treatment initiation.

**Conclusion:**

Agalsidase beta treatment preserves kidney function and delays progression to severe clinical events among adult patients with FD. Plasma GL-3 levels analysed in paediatric patients showed normalization of elevated pre-treatment levels in most patients.

## Graphical Abstract

**Graphical Abstract:**
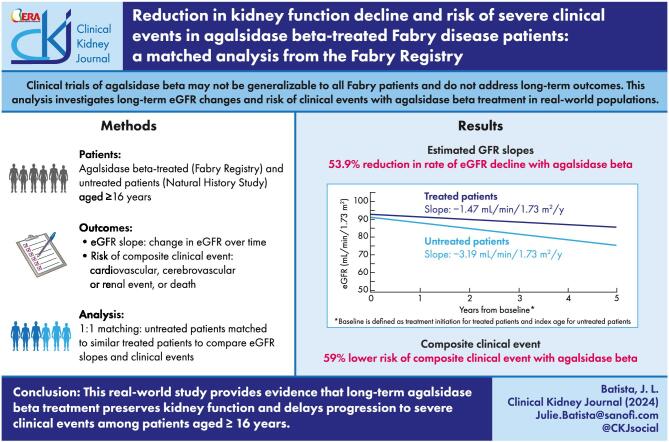

KEY LEARNING POINTS **What was known:**

- Clinical evidence in applications that sought marketing authorization for enzyme replacement therapies for the rare lysosomal disorder Fabry disease were limited by relatively short study durations and low numbers of enrolled patients.
- The results of those studies may not be generalizable to all Fabry patients because of restricted trial eligibility criteria.

**This study adds:**

- A substantially lower rate of eGFR decline was observed in adults with Fabry disease receiving agalsidase beta compared with matched untreated patients.
- Treatment was associated with lower risk of a composite clinical event over time.
- Most paediatric patients had durably normalized plasma GL-3 levels within 6 months of treatment initiation.

**Potential impact:**

- This evidence about the clinical effectiveness of agalsidase beta treatment (conservation of kidney function, delaying progression to severe clinical events) complements that from the registrational trials.
- The real-world evidence was used in the supplemental biologics license application to the Food and Drug Adminstration to support full approval of agalsidase beta (granted in 2021).

## INTRODUCTION

Fabry disease (FD, OMIM #301 500) is an X-linked lysosomal disorder caused by pathogenic *GLA* gene variants [1, 2]. Deficiency of α-galactosidase A (α-Gal A) leads to progressive accumulation of glycosphingolipids, primarily globotriaosylceramide (GL-3/Gb3) within lysosomes [3–5], prominently in vascular endothelium, kidney cells, cardiomyocytes and peripheral neurons, which induces an insidious cascade of disease processes, including cellular injury, inflammatory responses, cell loss, fibrosis [2, 5, 6] and progressive multi-organ damage which can be fatal [2]. Globotriaosylsphingosine (lyso-GL-3/lyso-Gb3) levels may be markedly elevated in plasma, which have been associated with glomerular injury, inflammation, proliferation and growth of smooth muscle cells, fibrosis and sclerosis in *in vitro* studies [3, 6].

FD encompasses a spectrum of phenotypes, with the classic phenotype being the most severe and characterized by absent or markedly deficient α-Gal A activity, as seen in hemizygous males. Early symptoms and signs may include burning peripheral pain, episodic pain crises, cornea verticillata, angiokeratoma, gastrointestinal symptoms and impaired sweating [7]. Patients are progressively at risk of developing life-limiting vital organ complications including progressive chronic kidney disease leading to end-stage kidney disease [2, 8, 9] cardiomyopathy, myocardial fibrosis, arrhythmias [8, 10] and ischaemic stroke [11].

Patients with residual α-Gal A activity may develop a later-onset FD phenotype with onset of vital organ manifestations (predominantly cardiac and occasionally renal) in adulthood and slower progression than the classic phenotype [2, 8].

Female heterozygotes have a wide spectrum of clinical variability due to the type of *GLA* variant and random X-chromosome inactivation. Generally, female patients are less severely affected than males, but the phenotype can range from asymptomatic to as severe as their affected male relatives [2, 8, 12].

In 2001, the introduction of two enzyme replacement therapies (ERTs) administered intravenously every other week, agalsidase alfa (0.2 mg/kg body weight) [13] and agalsidase beta (1 mg/kg body weight) [14, 15], provided the first opportunity to address the underlying α-Gal A deficiency and associated clinical FD sequelae. Several studies demonstrated beneficial effects of ERT on GL-3 clearance, kidney function decline, particularly when initiated early, and progression to a composite clinical event in patients with advanced FD [16–20]. However, the overall clinical evidence in marketing authorization applications was limited by the relatively short study durations and low numbers of enrolled patients, due to the rarity of this disorder. Moreover, such study results may not be generalizable to all patients because of the restricted trial eligibility criteria. Therefore, a larger, longer and more inclusive comparison of treated versus an untreated cohorts matched on demographics and clinical characteristics was undertaken to assess outcomes in FD after agalsidase beta treatment initiation.

This study investigated the associations of long-term agalsidase beta treatment (Fabrazyme^®^, Sanofi) with estimated glomerular filtration rate (eGFR) changes over time and the risk of developing a composite clinical event in a matched analysis of treated and untreated adult patients with FD. Changes in plasma GL-3 levels in paediatric patients were also evaluated.

## MATERIALS AND METHODS

### Data sources

Two distinct datasets of patients with FD were used in this study. Data for adult patients aged ≥16 years (matched analysis) and paediatric patients aged 2 to <16 years (GL-3 analysis) treated with agalsidase beta up to 5 January 2018 were from the Sanofi-sponsored Fabry Registry (NCT00196742). This ongoing global, longitudinal, observational study, initiated in 2001, collects patient data with the primary objectives of defining the variability in the natural history and clinical characteristics across the disease spectrum and characterizing long-term treatment outcomes in a real-world setting [21]. Each site is independent and responsible for obtaining informed written consent from patients for contributing their health data to the Fabry Registry and their use/disclosure in analyses.

Data for untreated adult patients (matched analysis) were from the International Epidemiologic Study of the Natural History of FD (hereinafter referred to as the Natural History Study) [22], which created a historical cohort of patients with FD. Medical records from 1944 to 2002 for 447 patients from five countries were abstracted to characterize the natural history of FD, including the occurrence of renal, cardiac and cerebrovascular events, or death, after patients, guardians or next of kin consented to release of their medical records. Additional approval from local Institutional Review Boards was obtained, if applicable.

Both the Fabry Registry and the Natural History Study complied with all relevant ethical and Good Clinical Practice requirements.

All data are reported according to the Strengthening the Reporting of Observational Studies in Epidemiology (STROBE) statement.

### Inclusion of adult patients in the matched analysis and matching criteria

Adult Fabry Registry patients were eligible for inclusion in the matched analysis if they were aged ≥16 years at agalsidase beta treatment initiation as their first FD treatment, had a non-missing date of first treatment and had no renal event (chronic dialysis, kidney transplant) prior to treatment initiation. Adult Natural History Study patients were eligible for matched analysis after their FD symptom onset and as long as they remained untreated. Patients with benign *GLA* variants were excluded from both datasets. Additional exclusion criteria specific to the eGFR slope and time-to-event analyses are further described in the Supplementary data (Supplementary text: Methodology for the matching algorithm).

Treated and untreated adult patients were matched by sex, phenotype (classic; later-onset; other/unclassified/missing) and index age (±5 years). FD phenotype was based on the International Fabry Disease Genotype-Phenotype Database classification (dbFGP.org, version 15 August 2018; on file) and/or on α-Gal A activity (≤1.5 nmol/h/mL in plasma or ≤4 nmol/h/mg protein in leukocytes defined as classic phenotype). The index age was defined as the age of treatment initiation in the treated patients; untreated patients were eligible to be matched if they had developed FD symptoms by the index age of a treated patient. For the eGFR slope analysis only, patients were further matched on baseline eGFR (±5 mL/min/1.73 m^2^), with baseline as the period around treatment initiation for treated patients and the index age for untreated patients.

Since the Natural History Study had considerably fewer adult patients than the Fabry Registry, we used two approaches to creating matched populations: a 1:1 matching ratio and an X:X ratio. The 1:1 dataset contained each treated patient and one matched untreated control; each untreated patient was matched to a treated patient only once in this dataset, so the number of treated patients was limited by the number of eligible untreated patients. The X:X dataset included all eligible treated Fabry Registry patients with an untreated match in the Natural History Study, with some untreated patients serving as controls for more than one treated patient. The 1:1 dataset is a subset of the X:X dataset. Details of the matching algorithms are provided in the Supplementary data (Supplementary text: Methodology for the matching algorithm).

### Clinical outcomes in adult patients

The outcomes assessed in adult patients were annualized eGFR slope over 5 years, estimated from serum creatinine using the Chronic Kidney Disease Epidemiology Collaboration equation [23], and a composite event variable defined as the first post-baseline occurrence of any of the following events: renal [chronic dialysis (>40 days), kidney transplant], cardiovascular (congestive heart failure, atrial fibrillation, ventricular tachycardia or significant cardiac procedure), cerebrovascular (haemorrhagic or ischaemic stroke) or death from any cause.

### Statistical analysis in adult patients

For the eGFR slope analysis, linear mixed effects models (LMMs) were used to estimate annualized eGFR slopes, their 95% confidence intervals (CIs) and the associated *P*-values for the slope's difference from 0 (i.e. no change over time; *P*_from 0_). Models included fixed and random effects for the intercept and time to account for repeated measures over time, and were fit separately for treated and untreated patients. For the X:X-matched population, untreated patients were weighted by the inverse of their frequency in the dataset, as they could occur multiple times as controls.

To assess the difference in slopes between treated and untreated patients, models were fit including both groups of patients with a product term of follow-up time and treatment group. This was used to estimate the slope difference between groups, its 95% CI and the associated *P*-value for the between group difference (*P*_difference_). Models that included both treated and untreated patients were conditioned on matched pairs to account for matching.

All models used an unstructured covariance matrix and restricted maximum likelihood estimation. eGFR slopes were estimated for the overall study population, by sex and within the subset of classic phenotype patients.

Several sensitivity analyses were conducted by including additional fixed effects in the models for: baseline age, baseline eGFR, use of angiotensin-converting enzyme inhibitor (ACEi)/angiotensin-receptor blocker (ARB) during follow-up, or baseline urine protein concentration (0 to <30 mg/dL, 30 to <100 mg/dL, 100 to <300 mg/dL, ≥300 mg/dL, missing). LMMs were also stratified by ACEi/ARB use to evaluate potential effect modification.

For the time-to-composite event analysis, Kaplan–Meier curves by treatment status were generated in the 1:1- and X:X-matched populations. Cox proportional hazards regression (shared frailty models), conditioned on matched pairs, was used to estimate hazard ratios (HRs), 95% CIs and *P*-values for the association of agalsidase beta treatment with risk of the composite endpoint.

For both the eGFR slope and time-to-composite event analyses, patients were followed from the index age for up to 5 years for the eGFR analysis or to the date of last clinical assessment for the time-to-event analysis. If applicable, follow-up for treated patients was censored at the date of a switch to any alternative therapy, and follow-up for untreated patients was censored at the date of initiation of FD therapy.

A two-sided *P*-value of <.05 was considered statistically significant. Statistical analyses were performed using SAS statistical software 9.4 (SAS Institute Inc., Cary, NC, USA).

### Plasma GL-3 changes in agalsidase beta–treated paediatric patients

As GL-3 may be a better surrogate indicator of therapeutic response than eGFR in paediatric FD patients, analyses of plasma GL-3 levels over time were conducted in agalsidase beta–treated patients in the Fabry Registry, who initiated treatment from 2 to <8 or 8 to <16 years of age. Patients with a history of dialysis/kidney transplant before treatment initiation were excluded, as were patients with benign *GLA* variants. Patients with a baseline (−6 months to +4 weeks from treatment initiation) plasma GL-3 assessment and at least one follow-up measure at 6 (±3), 12 (±3) or 24 (±3) months were analysed. Percent normalized (plasma GL-3 ≤7.03 μg/mL) [24] was calculated overall and among the subset of patients with elevated plasma GL-3 (>7.03 μg/mL) at baseline. Plasma GL-3 assessments after treatment switch or after the age of 16 years were excluded from analyses.

## RESULTS

For the eGFR slope analysis in adult patients, 950 treated patients were matched to 122 unique untreated patients (59.4% male pairs) in the X:X dataset, with 122 matched pairs (72.1% male pairs) in the subset of patients in the 1:1 dataset (Supplementary data, Table S1). For the time-to-event analysis in adult patients, 1754 treated patients were matched to 233 unique untreated patients (54.4% male pairs) in the X:X dataset, with 233 matched pairs (70.4% male pairs) in the subset of patients in the 1:1 dataset (Supplementary data, Table S2). No treated later-onset phenotype patients were successfully matched to untreated patients in the Natural History Study, due to the small number of later-onset patients in the Natural History Study.

Characteristics of the untreated and treated study populations are shown in Table 1 and, by sex, in Supplementary data, Table S3. On average, first FD symptom(s) occurred in childhood or early adulthood, earlier in males than in females, and agalsidase beta treatment was initiated when patients were in their fourth or fifth decade of life (means ranging from 32.9 to 35.3 years and 38.1 to 44.1 years, for males and females, respectively).

**Table 1:** Baseline demographics and clinical characteristics of untreated and agalsidase beta–treated adult patients with FD for the eGFR slope and time-to-event analyses.

	eGFR slope analysis			Composite clinical event analysis		
	Matching based on age, sex, phenotype and baseline eGFR			Matching based on age, sex and phenotype		
Characteristic	Unique untreated patients, 1:1 matched^a^ (*n* = 122)	Treated patients, 1:1 matched (*n* = 122)	Treated patients, X:X matched (*n* = 950)	Unique untreated patients, 1:1 matched^a^ (*n* = 233)	Treated patients, 1:1 matched (*n* = 233)	Treated patients, X:X matched (*n* = 1754)
Sex, *n* (%)	*n* = 122	*n* = 122	*n* = 950	*n* = 233	*n* = 233	*n* = 1754
Male	88 (72.1)	88 (72.1)	564 (59.4)	164 (70.4)	164 (70.4)	954 (54.4)
Female	34 (27.9)	34 (27.9)	386 (40.6)	69 (29.6)	69 (29.6)	800 (45.6)
Ethnicity, *n* (%)	*n* = 122	*n* = 122	*n* = 950	*n* = 233	*n* = 233	*n* = 1754
Caucasian	109 (89.3)	102 (83.6)	799 (84.1)	201 (86.3)	177 (76.0)	1391 (79.3)
Non-Caucasian	8 (6.6)	8 (6.6)	63 (6.6)	18 (7.7)	23 (9.9)	164 (9.4)
Unknown/missing	5 (4.1)	12 (9.8)	88 (9.3)	14 (6.0)	33 (14.2)	199 (11.3)
FD phenotype^b^, *n* (%)	*n* = 122	*n* = 122	*n* = 950	*n* = 233	*n* = 233	*n* = 1754
Classic	103 (84.4)	103 (84.4)	853 (89.8)	198 (85.0)	198 (85.0)	1456 (83.0)
Other/unclassified/missing	19 (15.6)	19 (15.6)	97 (10.2)	35 (15.0)	35 (15.0)	298 (17.0)
Age at symptom onset, mean (SD), years	*n* = 122	*n* = 103	*n* = 731	*n* = 233	*n* = 177	*n* = 1281
	12.3 (10.1)	14.4 (12.2)	16.8 (14.0)	12.8 (10.3)	15.6 (12.7)	17.7 (14.4)
Age at diagnosis, mean (SD), years	*n* = 117	*n* = 121	*n* = 934	*n* = 233	*n* = 228	*n* = 1714
	25.1 (12.8)	26.8 (14.1)	32.0 (14.4)	24.4 (13.5)	28.7 (13.5)	32.1 (14.5)
Age at agalsidase beta initiation, mean (SD), years	NA	*n* = 122	*n* = 950	NA	*n* = 233	*n* = 1754
	NA	35.0 (10.3)	38.9 (11.8)	NA	34.7 (11.4)	38.3 (12.3)
Baseline eGFR^c^, mean (SD), mL/min/1.73 m^2^	*n* = 122	*n* = 122	*n* = 950	NA	NA	NA
	91.1 (29.5)	91.4 (29.0)	92.0 (28.0)	NA	NA	NA
Number of eGFR assessments per patient per year, mean (SD), #/patient/year	*n* = 122	*n* = 122	*n* = 950	NA	NA	NA
	2.2 (1.64)	2.1 (1.32)	2.2 (1.43)			
Baseline urinary protein concentration^c,d^ categories, *n* (%)	*n* = 69	*n* = 93	*n* = 718	NA	NA	NA
Negative (0 to <30 mg/dL)	33 (47.8)	56 (60.2)	436 (60.7)	NA	NA	NA
1+ (≥30 to <100 mg/dL)	17 (24.6)	26 (28.0)	172 (24.0)	NA	NA	NA
2+ (≥100 to <300 mg/dL)	10 (14.5)	10 (10.8)	89 (12.4)	NA	NA	NA
3 + or higher (≥300 mg/dL)	9 (13.0)	1 (1.1)	21 (2.9)	NA	NA	NA
ACEi/ARB use during follow-up, *n* (%)	*n* = 122	*n* = 122	*n* = 950	*n* = 233	*n* = 233	*n* = 1754
	34 (27.9)	79 (64.8)	544 (57.3)	54 (23.2)	112 (48.1)	834 (47.5)
Follow-up, mean (SD), years	*n* = 122	*n* = 122	*n* = 950	*n* = 233	*n* = 233	*n* = 1754
	2.8 (1.4)	4.1 (1.0)	3.4 (1.4)	2.6 (2.9)	5.8 (4.1)	5.1 (3.9)

a The same untreated patients were contained in the 1:1- and X:X-matched populations, with multiple occurrences of each untreated patient in the X:X-matched population.

b Predicted FD phenotype was defined by *GLA* variants according to the International Fabry Disease Genotype-Phenotype database and α-Gal A activity.

c Baseline for the treated patients was the eGFR or UPCR assessment date closest (±6 months) to the date of agalsidase beta initiation; baseline for untreated patients was the earliest eGFR or UPCR assessment date after matching based on age (±5 years) of the treated patient.

d Baseline urinary protein concentration source: 24-h urine protein, spot urine protein, 24-h urine albumin, spot urine albumin or dipstick urine protein.

NA: not applicable; SD: standard deviation; UPCR: urine protein-to-creatinine ratio.

In the 1:1 matched population, mean follow-up time was 2.8 years in untreated and 4.1 years in treated patients for the eGFR slope analysis, and 2.6 and 5.8 years, respectively, for the time-to-event analysis. In the eGFR slope analysis, untreated patients were less likely to have a baseline urinary protein concentration in the negative range (0 to <30 mg/dL) than treated patients (47.8% vs 60.2% for the 1:1 matched population). The mean number of eGFR assessments per patient per year was similar in untreated and treated patients (2.2 vs 2.1 assessments/patient/year for the 1:1-matched population).

### eGFR slope in adult patients

**Figure 1:**
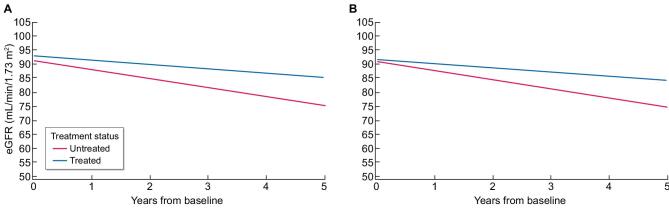
eGFR slopes in the overall populations of untreated and agalsidase beta–treated adult patients with FD. (**A**) Matching 1:1 based on age, sex, phenotype and baseline eGFR. Untreated: *n* = 122, eGFR slope −3.19 mL/min/1.73 m^2^/year; treated: *n* = 122, eGFR slope −1.47 mL/min/1.73 m^2^/year (both *P*_from 0_ < .001, *P*_difference_ = .007). (**B**) Matching X:X based on age, sex, phenotype and baseline eGFR. Untreated: *n* = 122, eGFR slope −3.29 mL/min/1.73 m^2^/year; treated: *n* = 950, eGFR slope −1.56 mL/min/1.73 m^2^/year (both *P*_from 0_ < .001, *P*_difference_ <.001). *P*_from 0_ is the *P*-value calculated to test whether the slope is different from 0. *P*_difference_ is the *P*-value comparing the slopes between groups.

Results of eGFR slope analyses are presented in Table 2. For the 1:1-matched population, eGFR slopes during up to 5 years of follow-up for untreated and treated patients were −3.19 (95% CI −4.33, −2.05) and −1.47 (95% CI −2.18, −0.76) mL/min/1.73 m^2^/year, respectively (both *P*_from 0 _< .001) (Fig. 1A), corresponding to a 53.9% reduction in the rate of decline in treated compared with untreated patients. The slope difference was 1.74 (95% CI 0.48, 3.00) mL/min/1.73 m^2^/year (*P*_difference _= .007) based on the model including both groups of patients.

**Table 2:** eGFR slopes in untreated and agalsidase beta–treated adult patients with FD.

			Untreated patients		Treated patients		
Matching	Population	*N* untreated/treated	eGFR slope mL/min/1.73 m^2^/year (95% CI)	*P*_from 0_^a^	eGFR slope mL/min/1.73 m^2^/year (95% CI)	*P*_from 0_^a^	*P*_difference_^b^
Total population: matching based on age, sex, phenotype and baseline eGFR							
1:1	All	122/122	−3.19 (−4.33, −2.05)	<.001	−1.47 (−2.18, −0.76)	<.001	.007
	Males	88/88	−3.93 (−5.31, −2.55)	<.001	−2.10 (−2.96, −1.25)	<.001	.019
	Females	34/34	−1.85 (−3.82, 0.12)	.065	0.36 (−0.73, 1.45)	.505	.027
X:X	All	122^c^/950	−3.29 (−3.75, −2.83)	<.001	−1.56 (−1.85, −1.27)	<.001	<.001
	Males	88^c^/564	−4.41 (−4.98, −3.85)	<.001	−2.31 (−2.70, −1.92)	<.001	<.001
	Females	34^c^/386	−1.16 (−1.81, −0.51)	<.001	−0.40 (−0.79, −0.02)	.042	.269
Patients with the classic phenotype^d^: matching based on age, sex, phenotype and baseline eGFR							
1:1	All	103/103	−3.04 (−4.32, −1.77)	<.001	−1.62 (−2.38, −0.86)	<.001	.040
	Males	81/81	−3.65 (−5.07, −2.24)	<.001	−2.13 (−3.00, −1.26)	<.001	.055
	Females	22/22	−0.99 (−3.72, 1.73)	.457	0.32 (−1.06, 1.69)	.636	.313
X:X	All	103^c^/853	−3.17 (−3.66, −2.68)	<.001	−1.62 (−1.92, −1.32)	<.001	<.001
	Males	81^c^/525	−4.12 (−4.71, −3.53)	<.001	−2.31 (−2.70, −1.92)	<.001	<.001
	Females	22^c^/328	−0.70 (−1.35, −0.04)	.039	−0.44 (−0.85, −0.02)	.040	.797

a *P*-value to test whether the slope was different from 0.

b *P*-values to compare the slopes between groups.

c Denoted number of unique untreated patients, but each may be matched to multiple treated patients; weights were applied to the model to account for this.

d Predicted FD phenotype is defined by *GLA* variants according to the International Fabry Disease Genotype-Phenotype database and α-Gal A activity.

For the X:X-matched population, slopes in untreated and treated patients were −3.29 (95% CI −3.75, −2.83) and −1.56 (95% CI −1.85, −1.27) mL/min/1.73 m^2^/year, respectively (both *P*_from 0 _< .001) (Fig. 1B), corresponding to a 52.6% reduction in the rate of decline for treated patients. The slope difference was 1.62 (95% CI 0.95, 2.29) mL/min/1.73 m^2^/year (*P*_difference _< .001).

In the 1:1-matched population, the rate of eGFR decline in untreated patients was more pronounced among the subsets of males [all males: −3.93 (95% CI −5.31, −2.55) mL/min/1.73  m^2^/year; classic males: −3.65 (95% CI −5.07, −2.24) mL/min/1.73 m^2^/year] than among all untreated patients combined [−3.19 (95% CI −4.33, −2.05) mL/min/1.73 m^2^/year]. A similar pattern was seen for the X:X-matched slopes. Results were inconsistent in the female subgroups, which included smaller numbers of patients.

The inclusion of baseline age, baseline eGFR, baseline urine protein concentration and ACEi/ARB use as fixed effects in the LMMs did not change the results of the eGFR slope analyses, and *P*_difference_ values remained statistically significant in the overall and male populations (Supplementary data, Table S4).

### Time to composite event in adult patients

**Figure 2:**
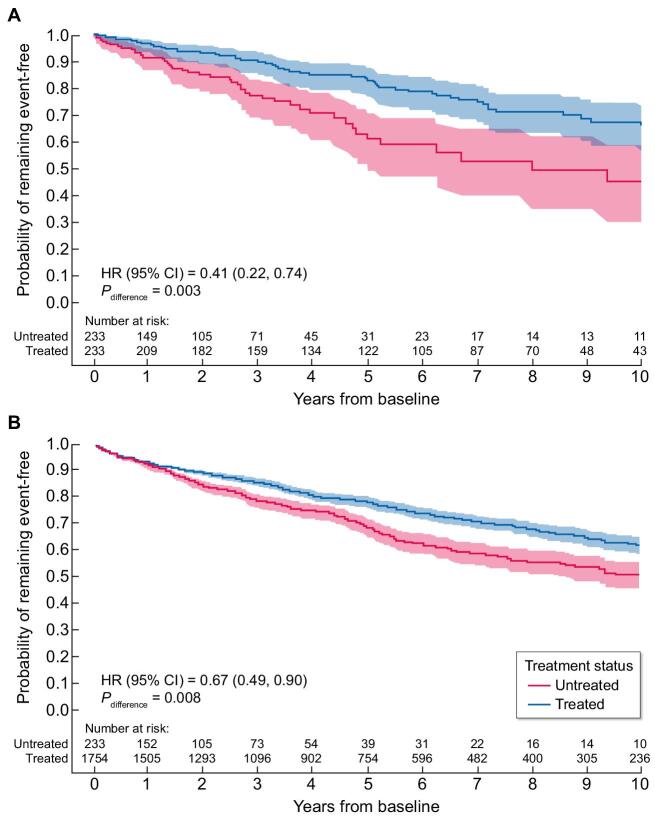
Time-to-composite clinical event in the overall populations of untreated and agalsidase beta–treated adult patients with FD. (**A**) Matching 1:1 by age, sex and phenotype. (**B**) Matching X:X by age, sex and phenotype. *P*_difference_ is the *P-*value from the Cox proportional hazards model comparing the risk between groups. Shading represents 95% Hall–Wellner bands.

Results of time-to-event analyses are presented in Table 3. Agalsidase beta–treated patients had a lower risk of experiencing a composite clinical event than untreated patients for both 1:1-matched (HR = 0.41; 95% CI 0.22, 0.74; *P* = .003; Fig. 2A) and X:X-matched populations (HR = 0.67; 95% CI 0.49, 0.90; *P* = .008; Fig. 2B). When stratified by sex, these associations were seen in males, but not in females.

**Table 3:** Time-to-event analysis showing incidence rates and HRs for the composite outcome in untreated and agalsidase beta–treated adult patients with FD.

Matching	Population	*N* untreated/treated	Incidence rate (per 1000 person-years) untreated/treated	HR (95% CI)	*P-*value^a^
All patients: matching based on age, sex and phenotype					
1:1	All	233/233	80.9/39.1	0.41 (0.22, 0.74)	.003
	Males	164/164	107.7/44.7	0.43 (0.23, 0.83)	.012
	Females	69/69	35.5/23.0	0.29 (0.06, 1.38)	.118
X:X	All	1754^b^/1754	69.2/49.2	0.67 (0.49, 0.90)	.008
	Males	954^b^/954	100.7/55.7	0.63 (0.45, 0.88)	.006
	Females	800^b^/800	34.2/40.0	0.97 (0.47, 1.98)	.923
Patients with the classic phenotype^c^: matching based on age, sex and phenotype					
1:1	All	198/198	74.3/42.9	0.54 (0.29, 1.00)	.051
	Males	149/149	91.6/48.5	0.57 (0.29, 1.12)	.100
	Females	49/49	38.9/23.3	0.40 (0.08, 2.06)	.273
X:X	All	1456^b^/1456	61.9/50.9	0.77 (0.54, 1.09)	.137
	Males	810^b^/810	88.0/56.8	0.76 (0.51, 1.12)	.168
	Females	646^b^/646	36.4/42.0	0.94 (0.41, 2.13)	.874

a *P*-values for the significance of the HR for the association of agalsidase beta treatment with the risk of the composite endpoint.

b The number of times untreated patients were matched to treated patients; untreated patients may be matched to multiple treated patients in the X:X analysis. A weighting scheme was applied to account for this fact when calculating the HR and associated *P*-value.

c Predicted FD phenotype was defined by *GLA* variants according to the International Fabry Disease Genotype-Phenotype database and α-Gal A activity.

Among classic patients, the magnitude and direction of the HRs for the risk of clinical events in treated versus untreated patients were comparable to the overall population (1:1 matched: HR = 0.54; 95% CI 0.29, 1.00; *P* = .051; X:X matched: HR = 0.77; 95% CI 0.54, 1.09; *P* = .137), although the results in this smaller subset of patients were not statistically significant.

In the 1:1-matched population, incidence rates of each component of the composite outcome were lower among treated versus untreated patients, except for cardiac and cerebrovascular events in females, which were based on a very low number of events (Supplementary data, Table S5). The most frequently reported events were renal (untreated: 46.2 per 1000 person-years; treated: 12.6 per 1000 person-years) and cardiovascular (untreated: 18.2 per 1000 person-years; treated: 16.2 per 1000 person-years). Percentages of patients reporting individual events are also presented, although these should be interpreted with caution as the length of observation was shorter in untreated than treated patients (606 versus 1354 total person-years) since the Natural History Study concluded shortly after agalsidase beta was approved for commercial use in 2001.

### Plasma GL-3 changes in agalsidase beta–treated paediatric patients

Demographic characteristics of the agalsidase beta–treated paediatric patient population included in the plasma GL-3 analysis (*n* = 100; 2 to <8 years, *n* = 29; 8 to <16 years, *n* = 71) are presented in Supplementary data, Table S6. At baseline, 60.0% of paediatric patients had plasma GL-3 levels above normal. Among these patients, plasma GL-3 fell within the normal range in 92.5%, 95.3% and 95.7% at 6, 12 and 24 months after treatment initiation, respectively (Supplementary data, Table S7). Results were similar between the two age groups. Only one female had a baseline plasma GL-3 above the normal level (which normalized during follow-up), thus limiting the plasma GL-3 results predominantly to male paediatric patients.

## DISCUSSION

This matched analysis of treated and untreated adult FD patients showed that long-term treatment with agalsidase beta was associated with both a significantly slower decline in kidney function and a lower risk of clinical events. Among paediatric patients, plasma GL-3 levels markedly declined within 6 months of agalsidase beta initiation and remained normalized through 24 months of follow-up.

In rare diseases such as FD, practical and ethical issues make it impossible to conduct large, randomized trials comparing long-term treatment with placebo. Previous efforts to quantify the impact of ERT on FD progression used data from two Fabry registries, the ongoing Fabry Registry [21] and the completed Fabry Outcome Survey [25], including meta-analyses of study data from individual patients [26] or from aggregate data from multiple studies [27–29]. The matched cohort design of the current study had the advantage of identifying untreated adult control patients individually matched with treated adult patients based on key baseline characteristics, thus providing a valid group for comparing long-term outcomes. The two different matching schemes (1:1 and X:X) provided reassurance of the validity of the results as both approaches revealed quite similar patterns of clinical benefit resulting from agalsidase beta treatment.

Fabry nephropathy is a prominent feature of FD characterized by progressive glomerular injury, proteinuria, podocyte damage and loss, glomerulosclerosis and kidney fibrosis [2, 30]. In patients with the classic phenotype, early and progressive GL-3 accumulation affects multiple kidney cells (e.g. vascular endothelial, glomerular, interstitial and tubular cells, and podocytes). Signs of occult kidney injury (including albuminuria and glomerulosclerosis) are often detected at a young age [31–33] and can lead to kidney dysfunction (e.g. progressive GFR decline, overt proteinuria) and kidney failure in adulthood [2, 8, 9]. In contrast, the reported rate of eGFR decline in the general adult population, beginning at age 40 years, is approximately −1 mL/min/1.73 m^2^/year [34].

In the current study, agalsidase beta treatment was associated with statistically significant slower decline in kidney function for up to 5 years (1:1 analysis: 53.9% reduction, slope difference 1.74 mL/min/1.73 m^2^/year; X:X analysis: 52.6% reduction, slope difference 1.62 mL/min/1.73 m^2^/year). This is clinically important since it plausibly delays or obviates the need for renal replacement therapy, which was the strongest driver of the overall clinical event rate in untreated patients in the current study. Significant differences were not observed in females, likely due to smaller numbers (and thus lower statistical power), along with wider variation in disease severity/presentation in classic and later-onset females, and markedly slower rates of disease progression, than classic and later-onset males [8]. Moreover, treatment was initiated relatively late in both female and male patients (in their late-30s or at older age, and around their mid-30s, respectively) whereas there is growing evidence that the clinical response to treatment is improved with early initiation [2, 35, 36]. Guidelines suggest that initiation should be considered in females with a *GLA* variant predictive of the classic FD phenotype if there are signs and/or symptoms suggesting major organ involvement of FD, and initiation in males with a classic *GLA* variant should be considered and is appropriate at any age of presentation [2].

Agalsidase beta treatment was also associated with a statistically significant lower incidence of composite clinical events, based on both 1:1 and X:X matching, in the overall adult patient population (59% and 33% reduction in risk, respectively) and in the male subgroup (57% and 43% reduction in risk, respectively). HRs were directionally consistent in the smaller subgroups of female and classic patients but were not statistically significant.

The results of the current analyses are consistent with those from other studies of agalsidase beta with ≤5 years of follow-up. In adult patients with classic FD (97% male) who participated in the open-label Phase 3 extension study, sustained treatment with agalsidase beta (median duration: 52.2 months) stabilized kidney function in 52 of 58 (89.7%) patients (mean eGFR decline −0.4 mL/min/1.73 m^2^/year), all of whom had low renal involvement at baseline (proteinuria ≤1 g/24 h and glomerular sclerosis <50%) [18]. Progressive decline in kidney function was observed in six (10.3%) patients, all of whom shared a common clinical profile of age >40 years, significant baseline proteinuria (>2 g/24 h) and >50% glomerular sclerosis at treatment baseline. In the Phase 4, randomized, placebo-controlled study that enrolled 82 adult patients (88% male) with advanced classic FD, the likelihood of any clinical event (composite outcome) in agalsidase beta–treated patients was markedly reduced compared with patients receiving placebo (median duration: 18.4 months), indicating a slower progression of severe manifestations [16]. An observational study from the Fabry Registry determined trends in the incidence of severe clinical events over time in adult patients with FD (641 males, 403 females, later-onset patients excluded) treated with agalsidase beta for up to 5 years. The incidence rate of combined events decreased after the first 6 months of treatment and remained stable thereafter [21, 37]. Patients at highest risk of events (males aged ≥40 years) displayed the greatest absolute reduction in incidence rate after the first 6 months of treatment [37].

Despite progressive GL-3 accumulation in plasma and kidneys beginning early in life, kidney function typically remains normal or near normal in young patients [33, 38], and may become abnormal from early adulthood onward [8]. Therefore, normalization of plasma GL-3 with agalsidase beta in the current analysis and a previous clinical study in paediatric patients [39] suggests that GL-3 may be a better surrogate indicator of therapeutic response in classic FD children, who constituted most of the treated paediatric cohort. In a study of plasma GL-3 response to agalsidase beta treatment in adult classic FD patients, all 29 treated patients (primarily males), who had an elevated mean plasma GL-3 level of 14.4 µg/mL at baseline, experienced normalization of plasma GL-3 levels in the Phase 3 clinical study of agalsidase beta [15]. In classic FD patients who participated in the extended Phase 3 or Phase 4 clinical studies of agalsidase beta, no statistically significant association was found between titres of IgG antibodies toward agalsidase beta and interference with reduction of plasma GL-3 levels during treatment [40]. Although preclinical and clinical studies have demonstrated that intracellular accumulation of GL-3 is associated with structural damage and functional loss in disease-related tissues [5] (e.g. the degree of podocyte GL-3 accumulation correlates directly with urinary protein excretion [31]), strict correlations between plasma GL-3 and clinical symptomatology of FD are to be confirmed.

Agalsidase beta was granted accelerated approval by the US Food and Drug Administration (FDA) in 2003 based on a surrogate endpoint likely to predict clinical benefit (i.e. tissue GL-3 clearance), with full licensing subject to the collection of real-world evidence to confirm clinical benefit. The current analysis was used in the supplemental biologics license application to the FDA to support full approval, which was granted in March 2021 [15]. To the authors’ knowledge, this is the first time that real-world evidence has been used as the main support to obtain full approval of a therapy for a lysosomal storage disorder.

The large number of investigative sites and the multinational nature of the Fabry Registry reflects the clinical management of patients with FD and the use of agalsidase beta in real-world clinical practice globally, thus supporting more generalizable findings. Additionally, the body of real-world data made it feasible to investigate long-term outcomes in a rare disease among a sizeable number of patients with FD, in contrast to smaller, shorter-duration clinical trials. The use of eGFR slopes as the primary outcome reduced the potential for subjective interpretation inherent in histologic analyses, related to possible tissue sampling variability and pathologist intra-/inter-reader variability. Despite the use of different serum creatinine assays across the investigative sites which likely introduces more variability in the estimated slopes, statistically significant and clinically meaningful associations were observed between agalsidase beta use and a slower decline of kidney function in FD. Matching based on baseline age, sex, baseline eGFR and FD phenotype minimized potential confounding effects of these key factors. The similarities in age at symptom onset and initial diagnosis and in the number of eGFR assessments per patient per year further support comparability between the treated and untreated cohorts.

## LIMITATIONS

Given the time periods when the Natural History Study and Fabry Registry were conducted, confounding by indication or by disease severity could be a concern. However, Fabry Registry patients who initiated treatment would likely have more severe disease manifestations than the general FD population, introducing a selection bias towards poorer outcomes with agalsidase beta treatment, thus underestimating the true association and the impact of the treatment. Additionally, regional variations in the clinical management of patients (treated and untreated) may have introduced some biases. Findings in the small female subgroups were not consistent with the overall group. A larger female cohort would be informative but is challenging due to the underrepresentation of agalsidase beta–treated female patients with FD in the Fabry Registry. We lacked sufficient data on plasma lyso-GL-3, a biomarker of FD for assessment of biochemical response, in the paediatric patients. Finally, residual confounding by factors with limited data availability in these observational studies, such as proteinuria or ACEi/ARB use, cannot be excluded.

## CONCLUSION

These analyses demonstrated a substantially lower rate of eGFR decline in adult patients with FD receiving agalsidase beta than that in matched untreated patients, as reported in the US Prescribing Information [15]. Moreover, they demonstrated a lower risk of a composite clinical event over time. Most paediatric patients had stable plasma GL-3 levels durably normalized within 6 months of treatment initiation. The findings of the current study provide real-world evidence (complementary to that from the registrational trials) that long-term agalsidase beta treatment conserves kidney function and delays progression to severe clinical events in adult patients with FD.

## Supplementary Material

sfae194_Supplemental_File

## Data Availability

Data are available on reasonable request (Sanofi's guidance on data sharing).
