# Correction to: Reduction in kidney function decline and risk of severe clinical events in agalsidase beta–treated Fabry disease patients: a matched analysis from the Fabry Registry

**DOI:** 10.1093/ckj/sfae304

**Published:** 2024-10-17

This is a correction to: Julie L Batista, Ali Hariri, Manish Maski, Susan Richards, Badari Gudivada, Lewis A Raynor, Elvira Ponce, Christoph Wanner, Robert J Desnick, Reduction in kidney function decline and risk of severe clinical events in agalsidase beta–treated Fabry disease patients: a matched analysis from the Fabry Registry, *Clinical Kidney Journal*, Volume 17, Issue 8, August 2024, sfae194, https://doi.org/10.1093/ckj/sfae194.

In the originally published version of this manuscript, the link to the accompanying video abstract was inadvertently omitted.

This has been corrected.

