# Supplementary material for: Reduction in kidney function decline and risk of severe clinical events in agalsidase beta–treated Fabry disease patients: a matched analysis from the Fabry Registry
Source: Clin Kidney J. 2024 Jul 2;17(8):sfae194. doi: 10.1093/ckj/sfae194 (PMC11320591; doi:10.1093/ckj/sfae194)
Supplement: sfae194_Supplemental_File [file sfae194_Supplemental_File.docx]

**Supplementary Data**

**Methodology for the matching algorithm**

To summarise the matching algorithm, agalsidase beta-treated adult patients from the Fabry Registry and untreated adult patients from the Natural History Study that met the inclusion and exclusion criteria were eligible for matching, where the treated patient’s age at initiation of agalsidase beta was referred to as the “index age” for matching purposes.

For the estimated glomerular filtration rate (eGFR) matching algorithm, all untreated patients with the same sex and phenotype as the treated patient, who had an eGFR measure after symptom onset made within ±5 years of the “index age” of the treated patient and whose value was within ±5 mL/min/1.73 m^2^, as well as at least 1 additional eGFR measure between 0.5 and 5 years from the index age, and did not have renal dialysis or transplant prior to the index age, were candidates for matching. The eGFR assessment date closest to the “index age” was considered baseline (time 0) for both treated and untreated patients, and for treated patients, the eGFR assessment had to be between within ± 0.5 years from the index age.

For the composite clinical event matching algorithm, all untreated adult patients with the same sex and phenotype as the treated adult patient, who had a symptom onset on or before the treated patient’s index age, who had follow-up for clinical outcomes in the Natural History Study after the treated patient’s index age, and did not have renal dialysis or transplant prior to the index age, were chosen as candidates to match to the treated patient. The treated patient’s index age was considered baseline (time 0) for both treated and untreated patients. Patients who experienced the same cardiovascular or cerebrovascular event at both pre-baseline and post-baseline were removed as candidates for matching.

For both the eGFR and composite clinical event matching algorithms, for the X:X-matched population, all untreated matched candidates for each treated patient were identified, and 1 untreated matched patient was randomly selected to create the matched pair. Because the number of eligible treated patients was greater than the number of untreated patients, untreated patients may have been matched to more than 1 treated patient. To ensure a wide representation of untreated patients, once an untreated patient was matched, that patient was assigned a lower probability to be matched to a subsequent treated patient. As a result, the X:X dataset included all eligible unique treated Registry patients with an untreated match in the Natural History Study.

The 1:1 dataset was created from the X:X match to contain only 1 occurrence of each untreated patient, along with the subset of treated patients that are their matched partners. For the eGFR matching algorithm, the occurrence of each untreated patient with the longest follow-up period, along with his/her treated match, was selected from the pool of available matched pairs in the X:X dataset to attempt to increase the comparability between the treated and untreated patients with respect to follow-up time. For the composite clinical event matching algorithm, a randomly matched pair was selected from the pool of matched pairs to form the 1:1 dataset.

**Supplementary data, Table S1:** Consort flow of the study population of untreated and agalsidase beta-treated adult patients with Fabry disease: eGFR slope.

| **Entry Criteria** | **N** |
| --- | --- |
| **Agalsidase beta-treated Fabry Registry patients** | |
| Starting population: agalsidase beta as first primary therapy and non-missing date of first treatment | 2636 |
| *GLA* variant not a benign variant^a^ | 2581 |
| No renal event (dialysis or transplant) prior to agalsidase beta initiation | 2394 |
| Started agalsidase beta ≥16 years | 2083 |
| At least 2 eGFR measurements and non-missing baseline eGFR | 1357 |
| At least 1 eGFR measurement between 6 months and 5 years of baseline | 979 |
| Matched to untreated group^b^ | 950 |
| Classic phenotype | 853 |
| Other^c^/unclassified/unknown phenotype | 97 |
| **Untreated Natural History Study patients** | |
| Starting population: entire Natural History Study | 447 |
| *GLA* variant not a benign variant^a^ | 447 |
| Known therapy status | 446 |
| Known symptom onset date | 263 |
| eGFR measurement post symptom onset and prior to therapy start | 244 |
| No renal event prior to at least 1 eGFR measurement | 236 |
| At least two consecutive eGFR measurements within 6 months to 5 years from each other | 138 |
| Unique matches to agalsidase beta-treated patients^b^ | 122 |
| Classic phenotype | 103 |
| Other^c^/unclassified/unknown phenotype | 19 |

^a^Benign *GLA* variants including p.Ala143Thr, p.Pro60Leu, p.Asp313Tyr, p.Arg118Cys, p.Thr385Ala, IVS0-10 C>T, or the complex haplotype IVS0-10 C>T/IVS4-16A>G/IVS6-22C>T.

^b^Matching based on age, sex, Fabry disease phenotype, and baseline eGFR. The predicted Fabry disease phenotype was defined by *GLA* variants according to the International Fabry Disease Genotype-Phenotype database and α-Gal A activity. There were no later-onset phenotype patients who were matched.

^c^“Other” does not include variants listed as later-onset phenotype in the International Fabry Disease Genotype-Phenotype database.

eGFR: estimated glomerular filtration rate.

**Supplementary data, Table S2:** Consort flow of the study population of untreated and agalsidase beta-treated adult patients with Fabry disease: composite clinical event population.

| **Entry criteria** | **N** |
| --- | --- |
| **Agalsidase beta-treated Fabry Registry patients** | |
| Starting population: agalsidase beta as first primary therapy and non-missing date of first treatment | 2636 |
| *GLA* variant not a benign variant^a^ | 2581 |
| No renal event (dialysis or transplant) prior to agalsidase beta initiation | 2394 |
| Started agalsidase beta ≥16 years | 2083 |
| No duplicate events prior to agalsidase beta initiation^b^ | 2027 |
| Follow-up time greater than 0 | 2013 |
| Matched to untreated patients^c^ | 1754 |
| Classic phenotype | 1456 |
| Other^d^/unclassified/unknown phenotype | 298 |
| **Untreated Natural History Study patients** | |
| Starting population: Entire Natural History Study population | 447 |
| *GLA* variant not a benign variant^a^ | 447 |
| Known therapy status | 446 |
| Known symptom onset date | 263 |
| Valid follow-up date | 252 |
| Unique matches to agalsidase beta-treated patients^c^ | 233 |
| Classic phenotype | 198 |
| Other^d^/unclassified/unknown phenotype | 35 |

^a^Benign *GLA* variants including p.Ala143Thr, p.Pro60Leu, p.Asp313Tyr, p.Arg118Cys, p.Thr385Ala, IVS0-10 C>T, or the complex haplotype IVS0-10 C>T/IVS4-16A>G/IVS6-22C>T.

^b^Excludes patients with pre-baseline cardiovascular or cerebrovascular event identical to the first post-baseline event.

^c^Matching based on age, sex and Fabry disease phenotype. The predicted Fabry disease phenotype was defined by *GLA* variants according to the International Fabry Disease Genotype-Phenotype database and α-Gal A activity. There were no later-onset phenotype patients who were matched.

^d^“Other” does not include variants listed as later-onset phenotype in the International Fabry Disease Genotype-Phenotype database.

**Supplementary data, Table S3:** Baseline demographics and clinical characteristics of untreated and agalsidase beta-treated adult male and female patients with Fabry disease for the eGFR slope and time-to-event analyses.

| **Male patients with Fabry disease** | | | | | | |
| --- | --- | --- | --- | --- | --- | --- |
| **Characteristic** | **eGFR slope analysis**  **Matching based on age, sex, phenotype and baseline eGFR** | | | **Composite clinical event analysis**  **Matching based on age, sex and phenotype** | | |
|  | **Unique untreated patients, 1:1 matched^a^**  **(n = 88)** | **Treated patients, 1:1 matched**  **(n = 88)** | **Treated patients, X:X matched**  **(n = 564)** | **Unique untreated patients,  1:1 matched^a^**  **(n = 164)** | **Treated patients, 1:1 matched**  **(n = 164)** | **Treated patients, X:X matched**  **(n = 954)** |
| Ethnicity | n = 88 | n = 88 | n = 564 | n = 164 | n = 164 | n = 954 |
| Caucasian, n (%) | 79 (89.8) | 73 (83.0) | 463 (82.1) | 143 (87.2) | 122 (74.4) | 751 (78.7) |
| Non-Caucasian, n (%) | 7 (8.0) | 4 (4.5) | 47 (8.3) | 15 (9.1) | 18 (11.0) | 96 (10.1) |
| Unknown/missing, n (%) | 2 (2.3) | 11 (12.5) | 54 (9.6) | 6 (3.7) | 24 (14.6) | 107 (11.2) |
| Fabry disease phenotype^b^ | n = 88 | n = 88 | n = 564 | n = 164 | n = 164 | n = 954 |
| Classic, n (%) | 81 (92.0) | 81 (92.0) | 525 (93.1) | 149 (90.9) | 149 (90.9) | 810 (84.9) |
| Other/unclassified/missing, n (%) | 7 (8.0) | 7 (8.0) | 39 (6.9) | 15 (9.1) | 15 (9.1) | 144 (15.1) |
| Age at symptom onset, mean (SD), years | n = 88 | n = 79 | n = 468 | n = 164 | n = 130 | n = 761 |
|  | 10.0 (8.1) | 14.0 (11.7) | 14.3 (12.4) | 10.6 (8.0) | 14.0 (11.4) | 14.9 (12.8) |
| Age at diagnosis, mean (SD), years | n = 87 | n = 88 | n = 560 | n = 159 | n = 161 | n = 940 |
|  | 24.3 (12.1) | 25.7 (13.7) | 29.0 (13.3) | 22.9 (12.9) | 27.3 (12.9) | 29.1 (13.4) |
| Age at agalsidase beta initiation, mean (SD), years | NA | n = 88 | n = 564 | NA | n = 164 | n = 954 |
|  | NA | 33.8 (10.0) | 35.3 (10.9) | NA | 32.9 (10.2) | 34.7 (11.1) |
| Baseline eGFR^d^, mean (SD), mL/min/1.73 m^2^ | n = 88 | n = 88 | n = 564 | NA | NA | NA |
|  | 91.1 (31.9) | 91.5 (31.3) | 92.3 (32.5) | NA | NA | NA |
| Number of eGFR assessments per patient per year, mean (SD), #/patient/year | n = 88 | n = 88 | n = 564 | NA | NA | NA |
|  | 2.4 (1.7) | 2.2 (1.4) | 2.3 (1.6) | NA | NA | NA |
| Baseline urinary protein concentration^c,d^ categories, n (%) | n = 49 | n = 65 | n = 438 | NA | NA | NA |
| Negative (0 to <30 mg/dL) | 23 (46.9) | 38 (58.5) | 235 (53.7) | NA | NA | NA |
| 1+ (≥30 to <100 mg/dL) | 13 (26.5) | 18 (27.7) | 119 (27.2) | NA | NA | NA |
| 2+ (≥100 to <300 mg/dL) | 7 (14.3) | 9 (13.8) | 68 (15.5) | NA | NA | NA |
| 3+ or higher (≥300 mg/dL) | 6 (12.2) | 0 | 16 (3.7) | NA | NA | NA |
| ACEi/ARB use during follow-up, n (%) | n = 88 | n = 88 | n = 564 | n = 164 | n = 164 | n = 954 |
|  | 26 (29.5) | 58 (65.9) | 333 (59.0) | 42 (25.6) | 82 (50.0) | 478 (50.1) |
| Follow-up, mean (SD), years | n = 88 | n = 88 | n = 564 | n = 164 | n = 164 | n = 954 |
|  | 2.7 (1.4) | 4.1 (1.0) | 3.5 (1.3) | 2.3 (2.6) | 6.1 (4.3) | 5.5 (4.3) |
| **Female patients with Fabry disease** | | | | | | |
| **Characteristic** | **eGFR slope analysis**  **Matching based on age, sex, phenotype and baseline eGFR** | | | **Composite clinical event analysis**  **Matching based on age, sex and phenotype** | | |
|  | **Unique untreated patients, 1:1 matched^a^**  **(n = 34)** | **Treated patients, 1:1 matched**  **(n = 34)** | **Treated patients, X:X matched**  **(n = 386)** | **Unique untreated patients,  1:1 matched^a^**  **(n = 69)** | **Treated patients, 1:1 matched**  **(n = 69)** | **Treated patients, X:X matched**  **(n = 800)** |
| Ethnicity | n = 34 | n = 34 | n = 386 | n = 69 | n = 69 | n = 800 |
| Caucasian, n (%) | 30 (88.2) | 29 (85.3) | 336 (87.0) | 58 (84.1) | 55 (79.7) | 640 (80.0) |
| Non-Caucasian, n (%) | 1 (2.9) | 4 (11.8) | 16 (4.1) | 3 (4.3) | 5 (7.2) | 68 (8.5) |
| Unknown/missing, n (%) | 3 (8.8) | 1 (2.9) | 34 (8.8) | 8 (11.6) | 9 (13.0) | 92 (11.5) |
| Fabry disease phenotype^b^ | n = 34 | n = 34 | n = 386 | n = 69 | n = 69 | n = 800 |
| Classic, n (%) | 22 (64.7) | 22 (64.7) | 328 (85.0) | 49 (71.0) | 49 (71.0) | 646 (80.8) |
| Other/unclassified/missing, n (%) | 12 (35.3) | 12 (35.3) | 58 (15.0) | 20 (29.0) | 20 (29.0) | 154 (19.3) |
| Age at symptom onset, mean (SD), years | n = 34 | n = 24 | n = 263 | n = 69 | n = 47 | n = 520 |
|  | 18.0 (12.3) | 15.9 (13.8) | 21.3 (15.7) | 17.8 (13.1) | 20.1 (15.0) | 21.7 (15.6) |
| Age at diagnosis, mean (SD), years | n = 30 | n = 33 | n = 374 | n = 64 | n = 67 | n = 774 |
|  | 27.4 (14.6) | 29.8 (14.8) | 36.5 (14.9) | 28.1 (14.4) | 31.9 (14.4) | 35.8 (14.8) |
| Age at agalsidase beta initiation, mean (SD), years | NA | n = 34 | n = 386 | NA | n = 69 | n = 800 |
|  | NA | 38.1 (10.7) | 44.1 (11.2) | NA | 38.8 (13.0) | 42.7 (12.3) |
| Baseline eGFR^d^, mean (SD), mL/min/1.73 m^2^ | n = 34 | n = 34 | n = 386 | NA | NA | NA |
|  | 91.1 (22.8) | 91.3 (22.6) | 91.6 (19.8) | NA | NA | NA |
| Number of eGFR assessments per patient per year, mean (SD), #/patient/year | n = 34 | n = 34 | n = 386 | NA | NA | NA |
|  | 1.8 (1.4) | 1.9 (1.0) | 2.0 (1.1) | NA | NA | NA |
| Baseline urinary protein concentration^c,d^ categories, n (%) | n = 20 | n = 28 | n = 280 | NA | NA | NA |
| Negative (0 to <30 mg/dL) | 10 (50.0) | 18 (64.3) | 201 (71.8) | NA | NA | NA |
| 1+ (≥30 to <100 mg/dL) | 4 (20.0) | 8 (28.6) | 53 (18.9) | NA | NA | NA |
| 2+ (≥100 to <300 mg/dL) | 3 (15.0) | 1 (3.6) | 21 (7.5) | NA | NA | NA |
| 3+ or higher (≥300 mg/dL) | 3 (15.0) | 1 (3.6) | 5 (1.8) | NA | NA | NA |
| ACEi/ARB use during follow-up, n (%) | n = 34 | n = 34 | n = 386 | n = 69 | n = 69 | n = 800 |
|  | 8 (23.5) | 21 (61.8) | 211 (54.7) | 12 (17.4) | 30 (43.5) | 356 (44.5) |
| Follow-up, mean (SD), years | n = 34 | n = 34 | n = 386 | n = 69 | n = 69 | n = 800 |
|  | 2.9 (1.4) | 4.1 (1.1) | 3.2 (1.4) | 3.3 (3.5) | 5.0 (3.5) | 4.6 (3.4) |

^a^The same untreated patients were contained in the 1:1- and X:X-matched populations, with multiple occurrences of each untreated patient in the X:X-matched population.

^b^Predicted Fabry disease phenotype was defined by *GLA* variants according to the International Fabry Disease Genotype-Phenotype database and α-Gal A activity.

^c^Baseline for the treated patients was the eGFR or UPCR assessment date closest (−/+ 6 months) to the date of agalsidase beta initiation; baseline for untreated patients was the earliest eGFR or UPCR assessment date after matching based on age (−/+5 years) of the treated patient.

^d^Baseline urinary protein concentration source: 24-hour urine protein, spot urine protein, 24-hour urine albumin, spot urine albumin, or dipstick urine protein.

ACEi: angiotensin-converting enzyme inhibitor; ARB: angiotensin-receptor blocker; α-Gal A: α-galactosidase A; eGFR: estimated glomerular filtration rate; NA: not applicable; SD: standard deviation; UPCR: urine protein-to-creatinine ratio.

**Supplementary data, Table S4:** eGFR slopes in untreated and agalsidase beta-treated adult patients with Fabry disease: results of linear mixed modelling including baseline covariate adjustment.

|  | | | **Untreated** | | **Treated** | |  |
| --- | --- | --- | --- | --- | --- | --- | --- |
| **Matching** | **Population** | **N untreated/**  **treated** | **eGFR slope, mL/min/1.73 m^2^/y (95% CI)** | ***P*_from 0_^a^** | **eGFR slope, mL/min/1.73 m^2^/y (95% CI)** | ***P*_from 0_^a^** | ***P*_difference_^b^** |
| **Models adjusted for baseline age (continuous) and baseline eGFR (continuous)** | | | | | | | |
| 1:1 | All | 122/122 | −3.19 (−4.33, −2.05) | <0.001 | −1.47 (−2.18, −0.76) | <0.001 | 0.007 |
|  | Males | 88/88 | −3.93 (−5.31, −2.55) | <0.001 | −2.10 (−2.96, −1.25) | <0.001 | 0.019 |
|  | Females | 34/34 | −1.85 (−3.82, 0.12) | 0.065 | 0.36 (−0.73, 1.45) | 0.505 | 0.027 |
| X:X | All | 122^c^/950 | −3.29 (−3.75, −2.83) | <0.001 | −1.56 (−1.85, −1.27) | <0.001 | <0.001 |
|  | Males | 88^c^/564 | −4.41 (−4.98, −3.85) | <0.001 | −2.31 (−2.70, −1.92) | <0.001 | <0.001 |
|  | Females | 34^c^/386 | −1.16 (−1.81, −0.51) | <0.001 | −0.40 (−0.79, −0.02) | 0.042 | 0.269 |
| **Models adjusted for baseline urine protein categories^d^** | | | | | | | |
| 1:1 | All | 122/122 | −3.20 (−4.34, −2.06) | <0.001 | −1.45 (−2.16, −0.75) | <0.001 | 0.006 |
|  | Males | 88/88 | −3.84 (−5.21, −2.47) | <0.001 | −2.10 (−2.95, −1.25) | <0.001 | 0.023 |
|  | Females | 34/34 | −1.79 (−3.72, 0.13) | 0.067 | 0.32 (−0.75, 1.40) | 0.545 | 0.027 |
| X:X | All | 122^c^/950 | −3.22 (−3.68, −2.76) | <0.001 | −1.57 (−1.86, −1.28) | <0.001 | <0.001 |
|  | Males | 88^c^/564 | −4.34 (−4.92, −3.76) | <0.001 | −2.31 (−2.69, −1.92) | <0.001 | <0.001 |
|  | Females | 34^c^/386 | −0.66 (−1.32, 0.00) | 0.049 | −0.41 (−0.80, −0.02) | 0.037 | 0.560 |
| **Models adjusted for ACEi/ARB use (yes, no/missing)** | | | | | | | |
| 1:1 | All | 122/122 | −3.22 (−4.36, −2.08) | <0.001 | −1.47 (−2.18, −0.76) | <0.001 | 0.007 |
|  | Males | 88/88 | −3.95 (−5.33, −2.57) | <0.001 | −2.11 (−2.96, −1.25) | <0.001 | 0.019 |
|  | Females | 34/34 | −1.80 (−3.79, 0.19) | 0.074 | 0.40 (−0.69, 1.48) | 0.465 | 0.023 |
| X:X | All | 122^c^/950 | −3.29 (−3.75, −2.83) | <0.001 | −1.58 (−1.87, −1.30) | <0.001 | <0.001 |
|  | Males | 88^c^/564 | −4.44 (−5.01, −3.87) | <0.001 | −2.33 (−2.72, −1.94) | <0.001 | <0.001 |
|  | Females | 34^c^/386 | −1.11 (−1.77, −0.46) | <0.001 | −0.40 (−0.79, −0.01) | 0.043 | 0.302 |
| **Models among patients who ever reported ACEi/ARB use during follow-up^e^** | | | | | | | |
| 1:1 | All | 19/19 | −3.89 (−6.30, −1.49) | 0.003 | −2.29 (−4.26, −0.32) | 0.025 | 0.206 |
| X:X | All | 34^c^/272 | −4.77 (−5.63, −3.92) | <0.001 | −1.35 (−1.82, −0.88) | <0.001 | <0.001 |
| **Models among patients who never reported ACEi/ARB use (including no/missing) during follow-up^e^** | | | | | | | |
| 1:1 | All | 28/28 | −3.24 (−5.50, −0.99) | 0.006 | −0.66 (−1.93, 0.62) | 0.299 | 0.029 |
| X:X | All | 81^c^/651 | −2.70 (−3.23, −2.18) | <0.001 | −1.64 (−2.01, −1.28) | <0.001 | 0.019 |

^a^*P* value to test whether the slope was different from 0.

^b^*P* value to compare the slopes between groups.

^c^Denoted the number of unique untreated patients, but each may be matched to multiple treated patients; weights are applied to the model to account for this.

^d^Baseline urinary protein categories: negative (0 to <30 mg/dL), 1+ (30 to <100 mg/dL), 2+ (100 to <300 mg/dL), 3+ or higher (≥300 mg/dL), missing.

^e^The stratified approach required concordance between treated and untreated matched pairs; for the ever ACEi/ARB users, both the treated and untreated patients in the matched pair ever indicated ACEi/ARB use; for the never ACEi/ARB users, both the treated and the untreated patient in the matched pair never (including no/missing) indicated ACEi/ARB use.

ACEi: angiotensin-converting enzyme inhibitor; ARB: angiotensin-receptor blocker; CI: confidence interval; eGFR: estimated glomerular filtration rate.

**Supplementary data, Table S5:** Frequency and incidence rate of individual first-occurring clinical events in untreated and agalsidase beta-treated patients with Fabry disease, 1:1 matched population.

|  | **Untreated patients** | | | **Treated patients** | | |
| --- | --- | --- | --- | --- | --- | --- |
|  | **All** | **Males** | **Females** | **All** | **Males** | **Females** |
| N patients | 233 | 164 | 69 | 233 | 164 | 69 |
| Person-years | 606.0 | 380.6 | 225.5 | 1354.0 | 1006.1 | 348.0 |
| Any event |  |  |  |  |  |  |
| n events, % of patients | 49 (21.0) | 41 (25.0) | 8 (11.6) | 53 (22.7) | 45 (27.4) | 8 (11.6) |
| Incidence rate (per 1000 person-years) | 80.9 | 107.7 | 35.5 | 39.1 | 44.7 | 23.0 |
| Renal |  |  |  |  |  |  |
| n events, % of patients | 28 (12.0) | 24 (14.6) | 4 (5.8) | 17 (7.3) | 16 (9.8) | 1 (1.4) |
| Incidence rate (per 1000 person-years) | 46.2 | 63.1 | 17.7 | 12.6 | 15.9 | 2.9 |
| Cardiovascular |  |  |  |  |  |  |
| n events, % of patients | 11 (4.7) | 9 (5.5) | 2 (2.9) | 22 (9.4) | 18 (11.0) | 4 (5.8) |
| Incidence rate (per 1000 person-years) | 18.2 | 23.6 | 8.9 | 16.2 | 17.9 | 11.5 |
| Cerebrovascular |  |  |  |  |  |  |
| n events, % of patients | 7 (3.0) | 6 (3.7) | 1 (1.4) | 10 (4.3) | 7 (4.3) | 3 (4.3) |
| Incidence rate (per 1000 person-years) | 11.6 | 15.8 | 4.4 | 7.4 | 7.0 | 8.6 |
| Death |  |  |  |  |  |  |
| n events, % | 3 (1.3) | 2 (1.2) | 1 (1.4) | 4 (1.7) | 4 (2.4) | 0 (0.0) |
| Incidence rate (per 1000 person-years) | 5.0 | 5.3 | 4.4 | 3.0 | 4.0 | 0.0 |

**Supplementary data, Table S6:** Baseline demographics and clinical characteristics by age at treatment initiation among agalsidase beta-treated paediatric patients with Fabry disease who had baseline and ≥1 follow-up measurements of plasma GL-3^a^.

| **Age Group** | **2 to <8 years**  **(n = 29)** | **8 to <16 years**  **(n = 71)** | **Total**  **(N = 100)** |
| --- | --- | --- | --- |
| Sex | n = 29 | n = 71 | n = 100 |
| Male, n (%) | 27 (93.1) | 43 (60.6) | 70 (70.0) |
| Female, n (%) | 2 (6.9) | 28 (38.9) | 30 (29.7) |
| Ethnicity | n = 29 | n = 71 | n = 100 |
| Caucasian, n (%) | 20 (69.0) | 51 (71.8) | 71 (71.0) |
| Non-Caucasian, n (%) | 7 (24.1) | 10 (14.1) | 17 (17.0) |
| Unknown/missing, n (%) | 2 (6.9) | 10 (14.1) | 12 (12.0) |
| Fabry disease phenotype | n = 29 | n = 71 | n = 100 |
| Classic, n (%) | 26 (89.7) | 63 (88.7) | 89 (89.0) |
| Later-onset, n (%) | 2 (6.9) | 4 (5.6) | 6 (6.0) |
| Unspecified/unclassified/missing, n (%) | 1 (3.4) | 3 (4.2) | 4 (4.0) |
| Age at symptom onset, mean (SD), years | n = 19 | n = 49 | n = 68 |
|  | 3.6 (1.7) | 7.1 (3.4) | 6.1 (3.4) |
| Age at diagnosis, mean (SD), years | n = 29 | n = 71 | n = 100 |
|  | 3.3 (2.2) | 7.9 (4.1) | 6.6 (4.2) |
| Age at agalsidase beta initiation, mean (SD), years | n = 29 | n = 71 | n = 100 |
|  | 5.9 (1.5) | 12.3 (1.9) | 10.4 (3.4) |

^a^Baseline is a +4 week/−6 month window around agalsidase beta treatment initiation. The closest pre-treatment GL-3 measure, or closest post-treatment GL-3 measure is preferentially chosen. Follow-up is the closest value to the time point within a window of +/−3 months.

GL-3: globotriaosylceramide.

**Supplementary data, Table S7:** Changes in plasma GL-3 levels by age at treatment initiation among agalsidase beta-treated paediatric patients with Fabry disease.

|  | **All Paediatric Patients** | | | **Males** | | | **Females** | | |
| --- | --- | --- | --- | --- | --- | --- | --- | --- | --- |
| **Age Group** | **2 to <8 years** | **8 to <16 years** | **Total** | **2 to <8 years** | **8 to <16 years** | **Total males** | **2 to <8 years** | **8 to <16 years** | **Total females** |
| Baseline^a^ plasma GL-3 | | | | | | | | | |
| n | 29 | 71 | 100 | 27 | 43 | 70 | 2 | 28 | 30 |
| Median, µg/mL  Min, max; µg/mL | 10.5  3.1, 21.9 | 7.1  2.3, 21.7 | 8.4  2.3, 21.9 | 10.6  3.3, 21.9 | 9.7  3.3, 21.7 | 10.4  3.3, 21.9 | 3.2  3.1, 3.2 | 3.4  2.3, 7.4 | 3.3  2.3, 7.4 |
| Above normal (>7.03 µg/mL), n (%) | 24 (82.8) | 36 (50.7) | 60 (60.0) | 24 (88.9) | 35 (81.4) | 59 (84.3) | 0 | 1 (3.6) | 1 (3.3) |
| Plasma GL-3 categories among patients with above normal plasma GL-3 (>7.03 µg/mL) at baseline^b^ | | | | | | | | | |
| n | 24 | 36 | 60 | 24 | 35 | 59 | 0 | 1 | 1 |
| At 6 months, n | 22 | 31 | 53 | 22 | 30 | 52 | 0 | 1 | 1 |
| Normal, n (%) | 20 (90.9) | 29 (93.5) | 49 (92.5) | 20 (90.9) | 28 (93.3) | 48 (92.3) | 0 | 1 (100.0) | 1 (100.0) |
| Above normal, n (%) | 2 (9.1) | 2 (6.5) | 4 (7.5) | 2 (9.1) | 2 (6.7) | 4 (7.7) | 0 | 0 | 0 |
| At 12 months, n | 19 | 24 | 43 | 19 | 23 | 42 | 0 | 1 | 1 |
| Normal, n (%) | 18 (94.7) | 23 (95.8) | 41 (95.3) | 18 (94.7) | 22 (95.7) | 40 (95.2) | 0 | 1 (100.0) | 1 (100.0) |
| Above normal, n (%) | 1 (5.3) | 1 (4.2) | 2 (4.7) | 1 (5.3) | 1 (4.3) | 2 (4.8) | 0 | 0 | 0 |
| At 24 months, n | 13 | 10 | 23 | 13 | 9 | 22 | 0 | 1 | 1 |
| Normal, n (%) | 12 (92.3) | 10 (100.0) | 22 (95.7) | 12 (92.3) | 9 (100.0) | 21 (95.5) | 0 | 1 (100.0) | 1 (100.0) |
| Above normal, n (%) | 1 (7.7) | 0 | 1 (4.3) | 1 (7.7) | 0 | 1 (4.5) | 0 | 0 | 0 |

^a^Baseline is a +4 week/−6 month window around agalsidase beta treatment initiation. The closest pre-treatment GL-3 measure, or closest post-treatment GL-3 measure was preferentially chosen.

^b^Follow-up was the closest value to the time point within a window of +/−3 months.

GL-3: globotriaosylceramide.
